# HISTORY OF JOB STRAIN AND RISK OF LATE-LIFE DEPENDENCY: A NATIONWIDE SWEDISH REGISTER-BASED STUDY

**DOI:** 10.1093/geroni/igac059.1927

**Published:** 2022-12-20

**Authors:** Ingemar Kåreholt, Charlotta Nilsen, Janne Agerholm, Susanne Kelfve, Jonas Wastesson, Kirsten Nabe-Nielsen, Bettina Meinow

**Affiliations:** Jönköping University, Jönköping, Jonkopings Lan, Sweden; Jönköping University, Jönköping, Jonkopings Lan, Sweden; Karolinska Institutet, Stockholm, Stockholms Lan, Sweden; Linköping University, Linköping, Ostergotlands Lan, Sweden; Karolinska Institutet, Stockholm, Stockholms Lan, Sweden; University of Copenhagen, Copenhagen, Hovedstaden, Denmark; Stockholm Gerontology Research Center, Stockholm, Stockholms Lan, Sweden

## Abstract

There is substantial evidence that work plays a significant role in post-retirement health. Yet little is known about its role in when late-life dependency may occur. We examined associations between job strain and the risk of entering late-life dependency. Individually linked nationwide Swedish registers were used to identify people 70+ alive in January 2014, and who did not experience the outcome (late-life dependency) during two months prior to the start of the follow-up. Late-life dependency was operationalized as use of long-term care. Information about job strain was obtained via a job exposure matrice and matched with job titles. Cox regression models with age as time-scale (adjusted for living situation, educational attainment, country of birth, and sex) were conducted to estimate hazard ratios (HR) for entering late-life dependency during the 24 months of follow-up (n=993,595). Having an initial high starting point of job strain followed by an increasing trajectory throughout working life implied a 23% higher risk of entering late-life dependency at a younger age, compared with the reference group (low starting point with a decreasing trajectory). High initial starting point followed by a stable trajectory implied a 12% higher risk of entering late-life dependency at a younger age. High initial starting point followed by a decreasing trajectory implied a 10% risk reduction, and a low starting point with a stable trajectory implied a 22% risk reduction, of entering late-life dependency at a younger age. Reducing stressful jobs across working life may contribute to postponing late-life dependency.

